# Investigating Sociodemographic Factors and HIV Risk Behaviors Associated With Social Networking Among Adolescents in Soweto, South Africa: A Cross-Sectional Survey

**DOI:** 10.2196/publichealth.4885

**Published:** 2016-09-28

**Authors:** Janan Janine Dietrich, Fatima Laher, Stefanie Hornschuh, Busisiwe Nkala, Lucy Chimoyi, Kennedy Otwombe, Angela Kaida, Glenda Elisabeth Gray, Cari Miller

**Affiliations:** ^1^ Perinatal HIV Research Unit (PHRU) Faculty of Health Sciences University of the Witwatersrand Johannesburg South Africa; ^2^ Faculty of Health Sciences Simon Fraser University Vancouver, BC Canada

**Keywords:** mobile phone, adolescent health, HIV, health, social networking, mhealth, South Africa

## Abstract

**Background:**

Internet access via mobile phones and computers facilitates interaction and potential health communication among individuals through social networking. Many South African adolescents own mobile phones and can access social networks via apps.

**Objective:**

We investigated sociodemographic factors and HIV risk behaviors of adolescent social networking users in Soweto, South Africa.

**Methods:**

We conducted an interviewer-administered, cross-sectional survey of adolescents aged 14-19 years. Independent covariates of social networking were assessed by multivariate logistic regression analysis.

**Results:**

Of 830 adolescents, 57% (475/830) were females and the median age was found to be 18 years (interquartile range 17-18). Social networking was used by 60% of adolescents (494/830); more than half, that is, 87% (396/494) accessed social networks through mobile phones and 56% (275/494) spent more than 4 hours per day using their mobile phones. Social networking was independently associated with mobile usage 2-4 hours (adjusted odds ratio [AOR]: 3.06, CI: 1.69-5.51) and more than 4 hours per day (AOR: 6.16, CI: 3.46-10.9) and one (AOR: 3.35, CI: 1.79-6.27) or more sexual partner(s) (AOR: 2.58, CI: 1.05-6.36).

**Conclusions:**

Mobile phone–based social networking is prevalent among sexually active adolescents living in Soweto and may be used as an entry point for health promotion and initiation of low-cost adolescent health interventions.

## Introduction

Social networking offers the opportunity to offer low-cost, easily accessible information and interventions to reduce human immunodefiency virus (HIV) risk among adolescents [1]. Given the burden of HIV among young South Africans [2], novel uses of technology can be leveraged to disseminate information and interventions among adolescent social networking users [3]. Previous research conducted in developed settings show that HIV risk behaviors including depression, alcohol use, and risky sexual behaviors are associated with social networking usage [4-6]. Despite a large body of research conducted in South Africa about HIV risk behaviors among adolescents [2,7,8], very few researchers have specifically investigated the association between HIV risk behaviors and mobile phone–based social networking.

Understanding mobile phone–based social networking in populations of interest may help in the relevant delivery of health interventions. In South Africa, a low-to-middle-income country, population census data reveals that 95% of households own mobile phones, compared with only 19% that own computers, desktops, or laptops [9]. In studies of South African adolescents and young adults, 72-78% own mobile phones whereas 91% have access to one, and up to 84% access the Internet via mobile phones [10,11]. The lower cost of Internet access through mobile phone broadband packages compared with computer-based Internet access is an important factor in the growth of mobile Internet in South Africa [11]. Taken together, these data suggest that health interventions using mobile phone technologies can reach the majority of the population in South Africa.

Internet access via mobile phones and computers facilitates interaction and potential health communication among individuals through social networking. Adolescents in particular have integrated into their lives the daily use of social media, including text messaging, blogging, videos sites, and social networking. Social networking platforms allow users to create online profiles to interact with each other [12,13]. In developed settings, social networking has been used to assess online social networking use and patterns [14], recruit participants for research participation [15,16], distribute questionnaires [17], assess problematic social networking use [18], promote health (including sexual health) [19,20], engage young people into treatment and care of HIV [21], and investigate the association between social networking use and sexual risk behaviors [4-6].

At the time of data collection for this study, the available social networking platforms in South Africa, included Mxit, Facebook, Whatsapp, and Blackberry messaging. Mxit, a South African innovation, is an instant messaging app that allows sending and receiving messages privately and via online chat rooms, while being able to access games, movie clips, or download music. Since Mxit was launched in 2003, it has registered at least 38 million South African users, which would equate to 73% of the total population in South Africa. Of the users, 19% are 15 to 17 years and 47% are 18 to 25 years old. The main activities on social networking platforms include sending messages, inviting others to events and online groups, and following the activities of other users [11]. These activities suggest that messages disseminated on social networking platforms have the potential to snowball, which is a useful feature for optimizing the reach of HIV prevention interventions among young people. When one considers the penetration of social networking platforms in African settings, the relevance of leveraging them for health promotion interventions is evident [22].

A few studies, mainly from the developed world, have examined user characteristics of social networking platforms. The factors that have been investigated include demographics, sexual behavior, and substance use [23-25]. For example, a study among 1434 10^th^ grade students from 5 high schools in Southern California showed that higher levels of alcohol use were associated with sending and receiving friendship invitations, becoming friends with users that consume alcohol and also increasing alcohol use if their friends drank more. In addition, results revealed that exposure to risky online pictures posted by friends increased smoking behaviors amongst study participants [23]. Behavioral risk factors such as sexual partnerships and alcohol use have also been examined with respect to social networking platform use. Clayton et al (2013) indicated that alcohol use among undergraduate students from the United States was a predictor for emotional attachment to Facebook [26].

Developers in South Africa have attempted to harness mobile phone technology for health message delivery. For example, in 2008 LoveLife launched the first mobile social networking app in South Africa, called MyMsta, which was dedicated to youth empowerment and HIV prevention [27]. However, there is little published evidence on the effectiveness and cultural acceptability of these types of health interventions in developing world settings like South Africa, particularly among adolescents [22]. Adolescents in developing settings face the enormous challenge of the intersection between infectious and noninfectious disease epidemics in these countries. Understanding the characteristics of adolescents accessing social networking platforms in developing countries may lend insight into the development of appropriate and targeted health interventions. Few studies have specifically focused on the use of social networking among adolescents in South Africa and even fewer have published results of these studies. This study investigated demographic, socioeconomic, sexual behavior, alcohol use, depression, and mobile phone use associated with social networking amongst adolescents in Soweto, South Africa.

## Methods

### Study Design

This analysis forms part of the *Botsha Bophelo Adolescent Health Study (BBAHS)*, an interviewer-administered, cross-sectional survey of 830 adolescents aged 14-19 years living in Soweto.

### Setting

The study was conducted at the Perinatal HIV Research Unit (PHRU) and at Kganya Motsha Adolescent Centre (KMAC) in Kliptown. Kliptown is the oldest district in Soweto, and it comprises a mixture of purpose-built housing and informal housing known as shacks [28]. Soweto is a large peri-urban township of about 1.3 million official inhabitants [29]. KMAC was founded in 2008 as an adolescent-friendly HIV management centre serving the HIV voluntary counseling and testing (VCT) and sexual and reproductive health needs of adolescents aged 14-19 years [30].

### Participants

A targeted, stratified sampling approach was used based on geographic distribution across all areas of Soweto, as well as the sex and age of adolescents. Seven of the areas were informal areas (locally known as informal settlements), where adolescents lived in housing structures composed of metal structures (locally known as shacks), with shared access to water, toilets, and limited or no access to electricity. Eligibility criteria included age 14-19 years and living in Soweto. Participants were recruited from various locations within each area with the aim of ensuring representation from all areas of Soweto and recruiting hard-to-reach adolescents who may not have accessed health services. Research interviewers strategically targeted adolescents around malls, schools, and neighbourhood hangouts. Interviewers provided potential participants with recruitment flyers with contact information where interviewers could be reached. Interviewers were available on a dropin basis at KMAC and the PHRU or by appointment. To accommodate adolescent schedules, interview times were available after school and on the weekends. Some communities were more challenging to recruit from than others, particularly informal settlements where the visibility of adolescents was reduced due to the lack of community infrastructure for young people to congregate (ie, schools and malls). Participants learned about the study from research staff and were invited to follow-up if they were interested in participating. Participants were also invited to share the information about the study with their friends. Therefore, we have a convenience sample based on targeted recruitment strategies stratified by geographic location, age, and sex characteristics.

### Data Collection

Surveys were conducted between June 2010 and June 2012. Participants received a face-to-face interviewer-administered survey assessing demographics, mobile phone access and use, sexual risk behavior, and alcohol use. Trained, multilingual interviewers administered the survey in English or IsiZulu (the most commonly used local languages in Soweto) via an online Internet platform, Survey Monkey, using iPads or computers [16,31]. Each survey took about 60 minutes.

### Ethical Considerations

The ethics committees of the University of the Witwatersrand, Johannesburg, South Africa and Simon Fraser University, Vancouver, Canada approved study procedures. Participants received 50 ZAR (~ $7) for travel reimbursement. Written informed consent was obtained for all participants. In addition, participants younger than 18 years required written parental consent together with their own written assent.

### Measures

The primary outcome variable of interest in this analysis was social networking, defined as the use of social networking platforms such as Facebook, Twitter, and Mxit accessed through mobile phones. Social networking was measured by responses (yes vs no). Explanatory variables included demographics (including: gender, age, primary language spoken at home, education level [primary vs post primary]), socioeconomic (type of housing, source of spending money), sexual behavior (sexual orientation, sexually active, number of sexual partnerships, same sex partnerships, age at coital debut, condom use, intergenerational sex), alcohol and drug use in the last 6 months, depression, mobile phone use (eg, access to a mobile phone, access to the Internet, and source of Internet [computer vs mobile phone]), sexual partnerships ([none, one, and more than one], age at coital debut [less than 15 years, 15-16 years, and 17 or more years], intergenerational sex and condom use), and relational (eg, parental and adult presence in household) variables.

### Statistical Analysis

Participant characteristics of adolescents who accessed social networking sites are presented in frequencies and compared through bivariate analysis using chi-square and Fisher’s exact test. Variables with a significant association (*P*<.05) were considered for entry into the multivariate logistic regression model. Univariate and multivariate logistic regression were performed to determine crude and adjusted odds ratio (AOR), 95% confidence interval (CI), and their corresponding *P* values. Model fit was assessed using the Hosmer-Lemeshow goodness-of-fit statistic where the model was determined a good fit if the *P* value was not significant [32]. All statistical tests were two-sided and were conducted using STATA version 12.

## Results

### Demographic Characteristics

In total, 956 interviews were completed between 2010 and 2012. Following extensive data cleaning, a final sample of 830 adolescent participants was reached. Due to the nature of our recruitment strategy, we were not able to determine absolute response or refusal rates. The median age for the sample (n=830) was 18 years (IQR 17-18) and 57% (n=475) were female. Of all participants, 60% (n=494) used social networking platforms. The most commonly used platforms were Mxit (46%) and Facebook (34%).

Table 1 shows the comparison of characteristics of social networking users (n=494) to nonsocial networking users (n=336). In bivariate analyses, social networking was significantly associated with sex (*P*=.041), age group (*P*=.002), type of housing (*P*<.001), educational level (*P*<.001), access to mobile phone (*P*<.001), access to Internet (*P*<.001), and source of Internet (*P*<.001). Other variables associated with social networking were alcohol use in the past 6 months (*P*<.001), daily mobile phone usage (*P*<.001), presence of an adult in the household (*P*=.001), sexually active (*P*=.032), sexual partnerships (*P*=.011), and age at coital debut (*P*=.012).

**Table 1:** Summary profile of adolescents by social networking use (N=830), Soweto, South Africa.

Variable		N (830)	Social networking nonusers, n=336 (40.0%)	Social networking users, n=494 (60.0%)	*P* value
**Demographic**					
**Sex**					
	Male	355	158 (47.0)	197 (39.9)	.041
	Female	475	178 (53.0.)	297 (60.1)	
**Age group**					
	Less than 15 years	179	90 (27.5)	89 (18.0)	.002
	16-17 years	233	95 (29.1)	138 (28.0)	
	18-19 years	408	142 (43.4)	266 (54.0)	
**Language**					
	IsiZulu	446	186 (56.4)	260 (53.2)	.138
	IsiXhosa	75	36 (10.9)	39 (7.9)	
	Sesotho	120	39 (11.8)	81 (16.6)	
	Others	178	69 (20.9)	109 (22.3)	
**Education level**					
	Primary	198	101 (30.6)	97 (19.6)	<.001
	Post Primary	626	229 (69.4)	397 (80.4)	
**Socioeconomic**					
**Type of housing**					
	Formal	700	261 (78.4)	439 (89.8)	<.001
	Informal	122	72 (21.6)	50 (10.2)	
**Source of spending money**					
	Parents	647	254 (78.4)	392 (82.4)	.163
	Other (Employment, relatives, boy or girlfriend)	154	70 (21.6)	84 (17.6)	
**Sexual behavior**					
**Sexual orientation**					
	Heterosexual	686	267 (86.4)	419 (87.3)	.719
	Lesbian or gay or bisexual	103	42 (13.6)	61 (12.7)	
**Sexually active**					
	No	369	164 (48.8)	205 (41.6)	.032
	Yes	460	172 (51.2)	288 (58.4)	
**Sexual partnerships^a,b^**					
	None	70	36 (15.8)	34 (8.4)	.011
	One	366	130 (57.0)	236 (58.3)	
	More than one	197	62 (27.2)	135 (33.3)	
**Same sex partnerships^a,b^**					
	No	269	90 (90.9)	179 (95.7)	.101
	Yes	17	9 (9.1)	8 (4.3)	
**Age at coital debut^a,b^**					
	Less than 15 years	95	36 (25.2)	59 (23.1)	.012
	15-16 years	164	70 (49.0)	94 (36.9)	
	17 years or more	139	37 (25.9)	102 (40.0)	
**Condom use^a,b^**					
	Never	15	8 (8.9)	7 (3.9)	.161
	Sometimes	68	25 (27.8)	43 (24.0)	
	Always	186	57 (63.3)	129 (72.1)	
**Intergenerational sex^a,b^**					
	No	523	190 (87.6)	333 (84.3)	.275
	Yes	89	27 (12.4)	62 (15.7)	
**Alcohol and drug use in the last six months**					
**Alcohol use in the last six months**					
	No	294	150 (45.3)	144 (29.5)	<.001
	Yes	525	181 (54.7)	344 (70.5)	
**Drug use in the last six months**					
	No	714	331 (98.5)	483 (98.0)	.567
	Yes	15	5 (1.5)	10 (2.0)	
**Depression**					
	No	542	227 (67.6)	315 (63.9)	.276
	Yes	287	109 (32.4)	178 (36.1)	
**Mobile phone use**					
**Access to Internet**					
	No	253	214 (72.1)	39 (7.9)	<.001
	Yes	535	83 (28.0)	452 (92.1)	
**Source of Internet**					
	Computer	87	28 (32.6)	59 (13.0)	<.001
	Mobile phone	454	58 (67.4)	396 (87.0)	
**Access to mobile phones**					
	No	148	115 (38.1)	33 (6.7)	<.001
	Yes	648	187 (61.9)	461 (93.3)	
**Daily mobile phone usage**					
	0-1 hour	226	148 (49.8)	78 (15.9)	<.001
	2-4 hours	217	80 (26.9)	137 (28.0)	
	>4 hours	344	69 (23.3)	275 (56.1)	
**Relational or parental**					
**Parental presence**					
	No parents	49	23 (6.8)	26 (5.3)	.085
	One parent	296	132 (39.3)	164 (33.2)	
	Both parents	485	181 (53.9)	304 (61.5)	
**Presence of an adult in household**					
	No	125	67 (20.3)	58 (12.0)	.001
	Yes	696	263 (79.7)	427 (88.0)	

^a^Considers only those who responded to the item.

^b^Column proportions do not add up to 100% due to missing values in some variables.

### Characteristics Associated With Social Networking Use

The unadjusted and adjusted predictors of social networking use are presented in Table 2. In the adjusted logistic regression model, adolescents who used mobile phones for 2-4 hours (AOR: 2.89, CI: 1.80-4.65) or more than 5 hours (AOR: 5.99, CI: 3.79-9.48) daily and those with one (AOR: 1.75, CI: 1.04-2.94) or more than one (AOR: 2.64, CI: 1.40-4.96) sexual partner had a higher odds of social networking.

**Table 2:** Unadjusted and adjusted associations between selected variables and social networking among adolescents, Soweto (N=830).

Factor		Univariate			Multivariate		
		OR	95% CI	*P* value	AOR	95% CI	*P* value
**Sex**							
	Male	1.00 (Ref^a^)			-	-	-
	Female	1.34	1.01-1.77	.04	-	-	-
**Age group**							
	Less than 15 years	1.00 (Ref)					
	16-17 years	1.47	0.99-2.16	.06	-	-	-
	18-19 years	1.89	1.33-2.71	<.001	-	-	-
**Source of spending money**							
	Parents	1.00 (Ref)			-	-	-
	Others	0.77	0.55-1.11	.164	-	-	-
**Education level**							
	Primary	1.00 (Ref)					
	Post primary	1.85	1.31-2.49	<.001	-	-	-
**Type of housing**							
	Formal	1.00 (Ref)					
	Informal	0.41	0.23-0.61	<.001	-	-	-
**Presence of an adult in the household**							
	No	1.00 (Ref)					
	Yes	1.88	1.28-2.75	<.001	-	-	-
**Daily mobile phone usage**							
	0-1 hour	1.00 (Ref)			1.00 (Ref)		
	2-4 hours	3.27	2.22-4.83	<.001	2.89	1.80-4.65	<.001
	>4 hours	7.56	5.17-11.06	<.001	5.99	3.79-9.48	<.001
**Depressed**							
	No	1.00 (Ref)					
	Yes	1.18	0.88-1.58	.276	-	-	-
**Sexual partnerships**							
	None	1.00 (Ref)			1 (Ref)		
	One	1.92	1.15-3.22	0.01	1.75	1.04-2.94	.035
	More than One	2.31	1.32-4.02	<0.001	2.64	1.40-4.96	.003

^a^Ref: Reference group.

The unadjusted and adjusted predictors of social networking use by sex are presented in Table 3. In the adjusted logistic regression by sex, males whose source of spending money was not provided by parents (AOR: 0.58, CI: 0.34-0.99) had lower odds of social networking. Those who used mobile phones for 2-4 hours (AOR: 3.06, CI: 1.69-5.51) or more than 4 hours daily (AOR: 6.16, CI: 3.46-10.9) had a higher odds of social networking. Adolescent males with one (AOR: 3.35, CI: 1.79-6.27) or more than one (AOR: 2.58, CI: 1.05-6.36) sexual partner had a higher odds of networking. Among females, a higher odds of social networking was associated with spending 2-4 hours (AOR: 3.53, CI: 2.08-5.97) or more than 5 hours (AOR: 6.49, CI: 3.92-10.73) daily on mobile phones and having more than one sexual partner (AOR: 1.99, CI: 1.08-3.67).

**Table 3:** Unadjusted and adjusted associations between selected variables and social networking among adolescents by sex, Soweto.

		Males				Females			
Factor		Univariate		Multivariate		Univariate		Multivariate	
		OR (95% CI)	*P* value	AOR (95% CI)	*P* value	OR (95% CI)	*P* value	AOR (95% CI)	*P* value
**Age group**									
	Less than 15 years	1.00 (Ref^a^)				1.00 (Ref)			
	16-17 years	1.24 (0.69-2.23)	.463	-	-	161 (0.94-2.75)	.080	-	-
	18-19 years	1.82 (1.08-3.09)	.026	-	-	1.90 (1.16-3.09)	.010	-	-
**Type of Housing**									
	Formal	1.00 (Ref)				1.00 (Ref)			
	Informal	0.50 (0.28-0.92)	.025	-	-	0.36 (0.21-0.60)	<.001	-	-
**Education level**									
	Primary	1.00 (Ref)				1.00 (Ref)			
	Post Primary	1.56 (0.98-2.75)	.058	-	-	1.94 (1.23-3.07)	.004	-	-
**Source of spending money**									
	Parents	1.00 (Ref)		1.00 (Ref)		1.00 (Ref)		-	-
	Others	0.60 (0.36-1.00)	.052	0.58 (0.34-0.99)	.045	1.02 (0.62-1.68)	.939	-	-
**Daily mobile phone usage**									
	0-1 hour	1.00 (Ref)				1.00 (Ref)		1.00 (Ref)	
	2-4 hours	2.84 (1.60-5.06)	<.001	3.06 (1.69-5.51)	<.001	3.45 (2.03-5.86)	<.001	3.53 (2.08-5.97)	<.001
	>4 hours	6.24 (3.56-10.87)	<.001	6.16 (3.46-10.9)	<.001	8.74 (5.17-14.75)	<.001	6.49 (3.92-10.73)	<.001
**Sexual Partnerships**									
	None	1.00 (Ref)				1.00 (Ref)		1.00 (Ref)	
	One	1.61 (0.78-3.34)	.196	3.35 (1.79-6.27)	.036	2.28 (1.29-4.06)	.005	1.47 (0.91-2.38)	.12
	More than One	2.28 (1.08-4.84)	.031	2.58 (1.05-6.36)	.0002	4.94 (1.98-12.37)	.001	1.99 (1.08-3.67)	.027
**Presence of an adult in the household**									
	No	1.00 (Ref)				1.00 (Ref)			
	Yes	1.14 (0.64-2.03)	.653	-	-	2.77 (1.63-4.69)	<.001	-	-
**Depressed**									
	No	1.00 (Ref)				1.00 (Ref)			
	Yes	1.19 (0.76-1.87)	.450	-	-	1.13 (0.77-1.68)	.511	-	-

^a^Ref: Reference group.

## Discussion

### Principal Findings

This is one of the first studies to report on social networking use via mobile phones among adolescents in South Africa. The data show that social networking use among a sample of adolescents in Soweto was associated with their source of spending money from either parents or others, longer duration of daily mobile phone use, and having more than one sexual partnership. In an analysis by sex, social networking was associated in both sexes with longer daily use of mobile phones, being sexually active and having multiple sexual partners. Interestingly, for females, there was a relationship between social networking use and alcohol use. For males, social networking use was associated with longer daily use of mobile phones and having more than one sexual partner.

Despite Soweto being a typically lower socioeconomic community in South Africa, social network access was prevalent among 60% of adolescents, indicating the opportunity to introduce HIV risk reduction interventions via social networking platforms to young people in this setting. Mobile phones were the main point of social networking access for the majority of the adolescents. In developed settings like the United States, social networking has been shown to be a feasible tool for health research among adolescents [33]. Studies (Yonker et al, 2015) have also shown that using social networking among adolescents and young adults concerning their health choices has proven to be an essential medium of communication [34]. Magidson et al (2015) demonstrated the effective use of social networking to engage HIV positive adult men who have sex with men in care [35]. In sub-Saharan Africa, published literature on social networking among adolescent samples is limited.

In our study, we found that access to social networking was lower among adolescents with lower socioeconomic markers including less educational attainment, living in informal housing arrangements and no adult presence in the household. We also found that those adolescents accessing social networking were more likely to be older, sexually active, and using alcohol. Taken together, these findings indicate the excellent potential to reach adolescents in Soweto with HIV prevention interventions via social networking, particularly those with higher risk sexual behaviors including being sexually active, having more than one sexual partner, and those who use alcohol. However, it may be harder to reach adolescents facing lower socioeconomic hardships via social networking.

Among participants under age 18 years, almost a third reported alcohol use, although we did not measure the extent of use. Findings from the second South African youth risk behavior survey (2008) showed that half of school-going adolescents aged 13–19 years had ever used alcohol and a third having consumed alcohol in the past month. Two-thirds of social networking users reported alcohol use in the past 6 months. Of those who reported social networking, almost half were underage. Furthermore, half of female adolescents who consumed alcohol in the past 6 months were more likely to use social networking platforms [36].

The link between alcohol advertising and use among adolescents has been documented before; however, research conducted in the United States has shown conflicting evidence about the effect of media campaigns to reduce problematic alcohol use. There is evidence that links alcohol advertising with alcohol initiation [37]. Furthermore, there is evidence from the United States that exposure to friends’ accounts of alcohol use on social networking platforms could have an indirect effect on willingness to consume alcohol [38]. By extension it may be reasonable to assume that if adolescents were exposed to normative antialcohol interventions on social networking platforms, they may in turn become less likely to initiate or continue alcohol use. In this way, social networking platforms could allow targeting of specific populations for novel antialcohol interventions. Male adolescents are more likely to use alcohol at an early age but there has been an increase in the number of younger female adolescents consuming alcohol [39,40], which is a concerning trend because females are physiologically more at risk from the effects of alcohol use than males [41,42,43]. Young females are an emerging target market for alcohol producers [40]. Adolescent alcohol use has been described as a factor in female sexual assault and engaging in risky sexual behaviors [44,45]. Among social networking users, almost a third reported multiple sexual partnerships. Adolescents with multiple sexual partners are a higher risk group for sexually transmitted infections [2]. However, multiple and concurrent sexual partnering is a risk factor for HIV only if condom use is inconsistent and if a partner is infected with HIV [46]. In South Africa, multiple and concurrent sexual partnerships has been associated with HIV infection among young people [2,47-50]. Of sexually active young people aged 15-24 years in South Africa, 22% had more than one sexual partner in the past 12 months [47]. Social networking applications can potentially be used to promote positive health messages through adolescent social networks.

### Limitations

This study did not document the type of content that adolescents were accessing on the social networking platforms; therefore, we could not determine whether adolescents demonstrated health-seeking behaviors on these platforms. Social desirability bias may have affected variables, possibly underestimating self-reported risk behaviors. Social networking based health applications may not be broadly applicable but would be effective for subsets of the adolescent population who are able to obtain data bundles to access social networking sites. There were limitations to the survey items because we did not assess the level of alcohol use and concurrency of sexual partnerships.

### Conclusion

Our study suggests evidence about mobile phone–based social networking use among adolescents in Soweto, South Africa, and beyond. We recommend that future research addresses social networking platforms as a means of disseminating health interventions including positive alcohol and sexual behavior messaging among adolescent populations in South Africa.

## Abbreviations

HIV: human immunodefiency virus
AOR: adjusted odds ratio
